# Inhibition of BIRC2 Sensitizes α7-HPV-Related Cervical Squamous Cell Carcinoma to Chemotherapy

**DOI:** 10.3390/ijms222011020

**Published:** 2021-10-13

**Authors:** Chiao-Yun Lin, Chun-Chieh Wang, Ren-Chin Wu, Lan-Yan Yang, Chen-Bin Chang, Yu-Bin Pan, Angel Chao, Chyong-Huey Lai

**Affiliations:** 1 Department of Obstetrics and Gynecology, Chang Gung Memorial Hospital, Linkou Branch and Chang Gung University College of Medicine, Taoyuan 333, Taiwan; chiao.yun0101@gmail.com (C.-Y.L.); rilakumamon@gmail.com (C.-B.C.); Drangiechao@gmail.com (A.C.); 2 Gynecologic Cancer Research Center, Chang Gung Memorial Hospital, Linkou Branch Taoyuan, Taoyuan 333, Taiwan; jjwang@cgmh.org.tw (C.-C.W.); renchin.wu@gmail.com (R.-C.W.); 3 Departments of Radiation Oncology, Chang Gung Memorial Hospital, Linkou Branch Taoyuan, Taoyuan 333, Taiwan; 4 Department of Pathology, Chang Gung Memorial Hospital, Linkou Branch and Chang Gung University College of Medicine, Taoyuan 333, Taiwan; 5 Biostatics Unit, Clinical Trial Center, Chang Gung Memorial Hospital and Chang Gung University College of Medicine, Taoyuan 333, Taiwan; e8901145@gmail.com; 6 Clinical Informatics and Medical Statistics Research Center, Chang Gung University College of Medicine, Taoyuan 333, Taiwan

**Keywords:** cervical cancer, squamous cell carcinoma (SCC), α7-HPV, BIRC2, miR143-3p, LCL161

## Abstract

The α7-human papillomavirus (HPV)-related cervical squamous cell carcinoma (SCC) is associated with poor prognosis. We compared the genomic profiles of this disease in a cohort corresponding to the 2001–2014 period with various responses to radiotherapy or concurrent chemoradiation through microRNA (miR) profiling involving miR 4.0 array and human transcriptome array 2.0 analyses. A real-time quantitative polymerase chain reaction was then conducted to identify the predictive biomarkers. A significantly lower expression of miR143-3p in recurrent tumors (*p* = 0.0309) relative to that in nonrecurrent tumors was observed. The miR143-3p targeted the mRNA expression of the baculoviral inhibitor of the apoptosis protein (IAP) repeat-containing 2 (BIRC2; *p* = 0.0261). The BIRC2 protein levels (*p* = 0.0023) were significantly higher in recurrent tumors than in nonrecurrent tumors. Moreover, the miR-143-3p sensitized the response of α7-HPV-related cervical SCC to chemotherapy by targeting *BIRC2*. A combination of *BIRC2*-inhibitor LCL161 and topotecan exerted synergistic effects on cancer cells and animal tumor models. In a pooled cohort of α7-HPV-related cervical SCC (including mixed infections with non-α7-HPV) treated between 1993 and 2014, high *BIRC2* expression was associated with significantly worse outcomes (cancer-specific survival, hazard ratio (HR) = 1.42, *p* = 0.008; progression-free survival, HR = 1.64; *p* = 0.005). Summarily, BIRC2 constitutes a novel prognostic factor and therapeutic target for α7-HPV-related cervical SCC.

## 1. Introduction 

Cervical cancer is a leading cause of mortality in female malignancies worldwide [1]. Selecting the appropriate candidates for primary surgery and providing patients with postoperative adjuvant therapy according to risk of failure is crucial [2]. Radical surgery and radiotherapy (RT) are equally efficacious for early-stage invasive cervical cancer, whereas concurrent chemotherapy and RT (CCRT) remain the mainstay treatment for locally advanced diseases [2,3]. 

Human papillomavirus (HPV) infection is the etiologic agent of cervical intraepithelial lesions and invasive carcinoma [4,5]. With regard to the HPV genotyping, the results are prognostic for primary cervical cancer. Specifically, HPV-18 in patients with cervical cancer of stage I–IIA receiving primary surgery significantly predicts poor prognosis [2,6]. In 1067 patients with stage I–II International Federation of Gynecology and Obstetrics (FIGO) cervical cancer undergoing primary surgery, FIGO stage II, lymph node (LN) metastasis, parametrial extension, cervical stromal invasion depth of >1/3, grade 2/3, HPV-18 positivity, and an age of >45 years were determined to be independent prognostic factors [2]. α7-HPVs are predictors of poor outcomes in patients with locally advanced cervical cancer undergoing primary RT/CCRT [7]. In another study, among HPV18-positive and HPV58-positive patients, a significant improvement in survival was observed in the CCRT group relative to the RT-alone group, whereas no difference was observed among patients with HPV16-positive or HPV33-positive tumors in stage IIB with positive LN metastasis or stage III/IVA cervical SCC [8]. An investigation into the underlying molecular mechanisms revealed notable results.

A growing body of evidence suggests that microRNAs (miRs) have oncogenic potential, and aberrant miR expression has been identified in numerous types of human cancers [9,10]. In various cancers, in the acquisition of tumor cell resistance to conventional chemotherapy and novel biological agents, miRNAs were demonstrated to be dysregulated [11]. Because miRNAs are small and are well preserved in formalin-embedded archival tissues, the search for miR biomarkers has garnered a considerable amount of scholarly interest.

Herein, we observed that miR143-3p expression was significantly lower in recurrent tumors than in nonrecurrent tumors. Moreover, we identified a novel negative correlation between the mRNA expression of the baculoviral inhibitor of the apoptosis protein (IAP) repeat-containing 2 (BIRC2) and miR143-3p in patients with α7-HPV-related cervical SCC through analysis with miR 4.0 and human transcriptome array (HTA) 2.0 arrays. Increased levels of miR143-3p have been identified as tumor suppressing in multiple cancers [12,13], and Liu et al. [13] reported that long noncoding RNA HOTAIR regulates BCL-2 by targeting miR143-3p in cervical cancer. However, we discovered that miR-143-3p sensitizes the response of α7-HPV-related cervical SCC to chemotherapy by targeting BIRC2. Furthermore, high BIRC2 expression was associated with significantly worse outcomes (α7-HPV and multiple infections with non-α7-HPV). LCL161 is an inhibitor of BIRC2 and a mimetic of the second mitochondria-derived activator of caspases (SMAC). Several ongoing clinical trials are exploring the use of LCL161 as a therapeutic agent in the treatment of cancers other than cervical cancer [14]. Our results suggest that BIRC2 is a novel prognostic factor and therapeutic target for α7-HPV-related cervical SCC. Clinical trials using inhibitors of BIRC2 (SMAC antagonists) are warranted.

## 2. Results 

### 2.1. Patient Characteristics and Prognosis of HPV Genotype Groups

To elucidate the molecular profiles of tumor HPV genotypes in various responses (outcomes) to RT/CCRT and identify predictive biomarkers, we conducted a retrospective review of patients with cervical SCC (n = 1489) who received RT/CCRT at Chang Gung Memorial Hospital between 2001 and 2014. After the exclusion of patients who were undergoing primary surgery, belonged to nonsquamous cell carcinoma type, had no image data, or had no follow-up data, 972 patients eligible for HPV-genotyping analysis remained. Thereafter, another two patients were excluded for low-quality DNA, leaving 970 patients. they comprised patients with α7-HPV-related cervical SCC (n = 126), non-α7-HPV-related cervical SCC (n = 732), mixed cervical SCC (n = 76), and HPV-negative cervical SCC (n = 36; Supplementary Figure S1A). No significant between-group differences in cancer-specific survival (CSS; *p* = 0.104) were noted. By contrast, a significant difference (*p* < 0.001) was observed for progression-free survival (PFS) between the HPV-genotype groups (α7-HPV, non-α7-HPV, mixed, and negative; Supplementary Figure S2).

### 2.2. Lower miR-143-3p Expression in Cervical SCC with RT/CCRT Failure

**Figure 1:**
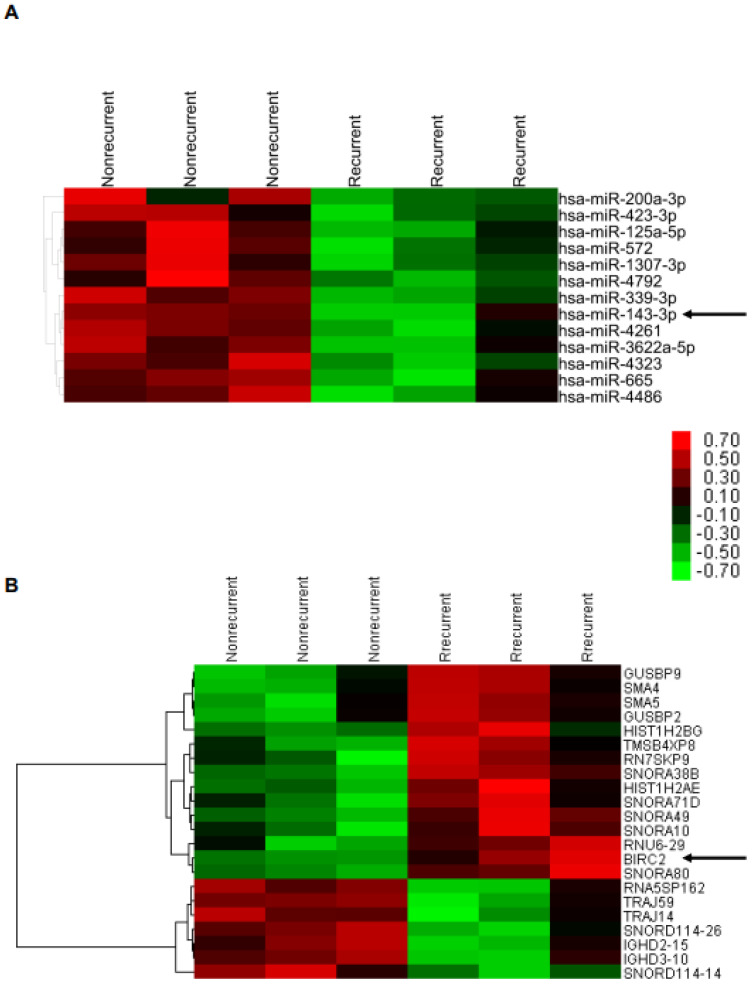
Identification of miRNA−mRNA regulatory relationships. (**A**) miR4.0 array and (**B**) HTA2.0 array analyses were performed on FFPE tissues of patients with recurrent and nonrecurrent cervical squamous cell carcinoma (SCC; n = 3 and 5, respectively).

From among the patients with α7-HPV-related SCC (n = 126), we selected formalin-fixed paraffin-embedded (FFPE) samples of patients with relatively less advanced disease (tumor size ≤ 5 cm, stage < 3, age < 80 years, n = 53 (recurrence/death, n = 14; nonrecurrence, n = 39)). We selected age- and grade-matched patients with recurrence/death (n = 3) and nonrecurrence (n = 3) for differentially expressed miR and mRNA profiling (Supplementary Figure S1B). We discovered a negative correlation between miR143-3p and *BIRC2*, its target gene, through both analyses of miR array 4.0 (*p* < 0.05, fold change [FC] > 4) and HTA 2.0 array (*p* < 0.05, FC > 2; Figure 1).

### 2.3. Higher BIRC2 mRNA Expression in α7-HPV-Related Cervical SCC 

**Figure 2:**
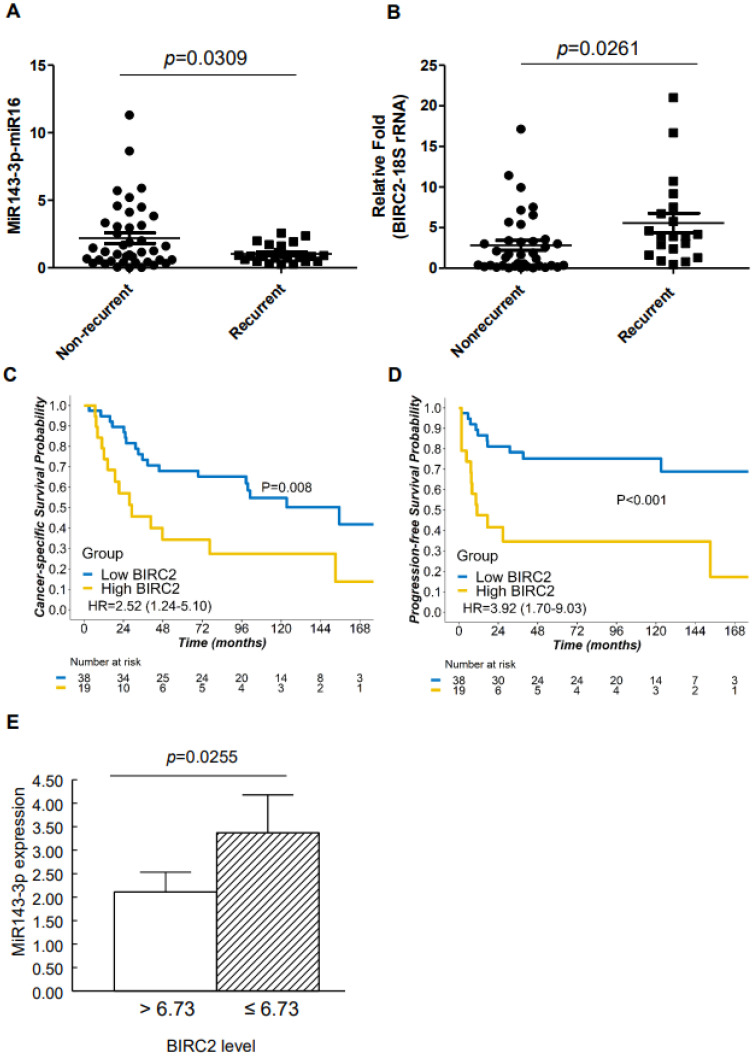
miR143-3p and *BIRC2* mRNA expression in α7-HPV-related SCC. (**A**,**B**) miR143-3p and BIRC2 were analyzed through real-time quantitative polymerase chain reaction (qPCR). (**C**,**D**) Kaplan–Meier plots of cancer-specific survival (CSS) and progression-free survival (PFS) in patients with cervical SCC. The optimal cutoff of expression for differentiating patients with cervical SCC who survived (n = 35) from those who did not (n = 22) was 3.734. The absolute number of patients at risk in each group at multiple time points is also reported. (**E**) The miR143-3p expression of the group with high *BIRC2* expression (>6.73, n = 10) was significantly lower than that of the group with low *BIRC2* expression (<6.73, n = 47).

We analyzed miR143-3p and *BIRC2* mRNA expression in FFPE tissue biopsy samples obtained from patients with α7-HPV-related cervical SCC through real-time quantitative polymerase chain reaction (qPCR). Because only 57 of the 126 patients (Supplementary Figure S1B) had adequate RNA quality for real-time qPCR, only these 57 samples were analyzed. The expression of miR143-3p was significantly lower in cervical SCC with recurrent tumors relative to that in nonrecurrent tumors (*p* = 0.0309; Figure 2A), whereas the mRNA expression of *BIRC2* was significantly higher in recurrent tumors than in nonrecurrent tumors (*p* = 0.0261; Figure 2B). Moreover, among patients with cervical SCC who were subsequently dichotomized according to *BIRC2* mRNA expression (cutoff = 3.734), those who exhibited higher *BIRC2* expression (n = 19) had less favorable CSS (hazard ratio (HR) = 2.52; 95% confidence interval (CI) = 1.24–5.10; *p* = 0.008; Figure 2C) and PFS (HR = 3.92; 95% CI = 1.70–9.03; *p* <0.001; Figure 2D) than did those with lower *BIRC2* expression (n = 38). The patients were categorized according to *BIRC2* mRNA expression as measured using cervical SCC specimens (n = 57), and the results were plotted against those of low versus high miR143-3p expression. The miR143-3p expression of the group with high *BIRC2* mRNA expression (>6.73) was significantly lower than that of the group with lower *BIRC2* mRNA expression (<6.73; Figure 2E).

### 2.4. Suppression of BIRC2 by miR143-3p in Cervical Cancer Cells 

**Figure 3:**
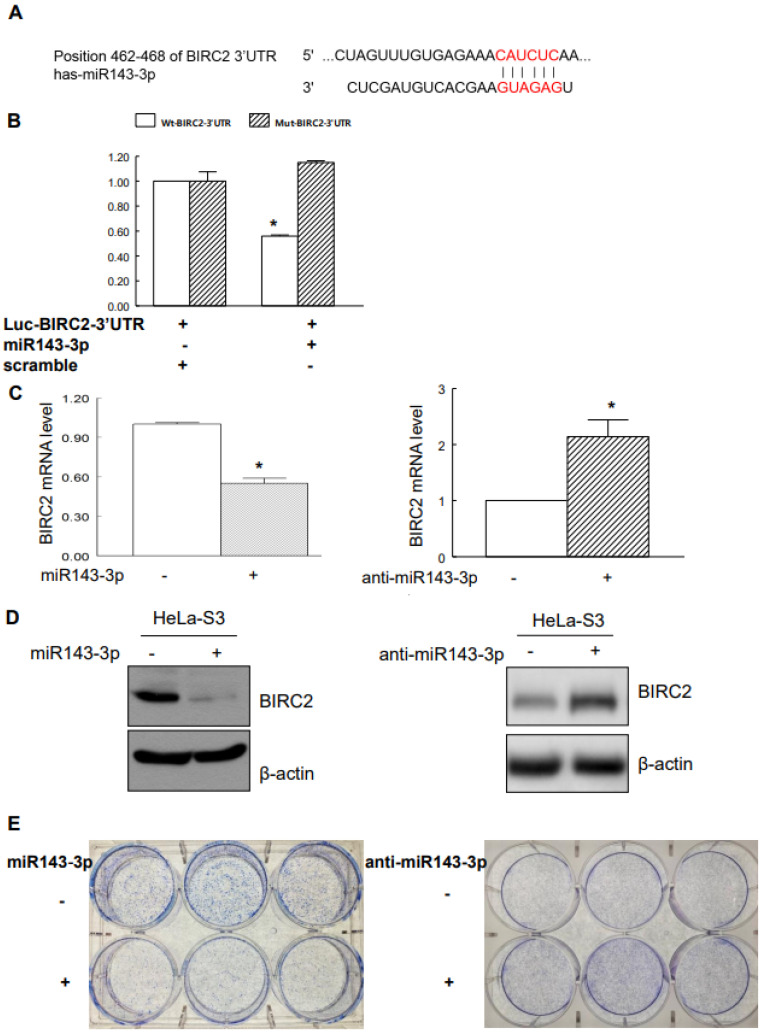
miR143-3p targeted *BIRC2* in cervical cancer cells. (**A**) Putative target site of miR143-3p at the 3′-UTR of BIRC2. (**B**) Effect of miR143-3p on the luciferase activity of wild-type Luc-BIRC2-3′-UTR (Wt-Luc-BIRC2-3′-UTR) and mut-Luc-BIRC2-3′-UTR (Mut-Luc-BIRC2-3′-UTR). HeLa-S3 cells were transfected with Luc-BIRC2-3′-UTR along with miR143-3p or scrambled as a control miR for 48 h. Data are presented as the means ± standard errors of relative luciferase activity, normalized to those of scrambled controls from three independent experiments. (**C**) Suppression of *BIRC2* mRNA through miR143-3p or transfection with anti-miR-143-3p increased mRNA expression of *BIRC2*, as detected through real-time qPCR conducted using BIRC2 and GAPDH primers. GAPDH was used as an internal control. (**D**) miR143-3p suppressed the expression of BIRC2 protein, and the suppression of endogenous miR-143-3p with anti-miR-143-3p increased BIRC2 protein levels (as determined through immunoblotting with β-actin as a loading control). (**E**) HeLa-S3 cells were transiently transfected with miR143-3p or anti-miR-143-3p for 7–14 days. Colony formation was analyzed through the clonogenic assay. * *p* < 0.05 relative to controls.

The targeting of *BIRC2* by miR143-3p was predicted using the miRBase and TargetScan databases (Figure 3A). To confirm that miR143-3p directly suppressed *BIRC2* expression through the target sequences of the 3′-UTR of BIRC2 mRNA, we constructed a pMiR luciferase reporter vector (Luc-BIRC2-3′-UTR) with the putative 3′-UTR target site for miR143-3p being located downstream from the luciferase gene. Cotransfected miR143-3p significantly reduced the reporter activity of Luc-BIRC2-3′-UTR but did not significantly affect the reporter activity of mut-Luc-BIRC2-3′-UTR (Figure 3B). To validate the role of miR143-3p in suppressing endogenous *BIRC2*, we overexpressed miR143-3p in HeLa-S3 cell, which significantly inhibited the endogenous *BIRC2* mRNA and protein levels. By contrast, the overexpression of anti-miR-143-3p significantly upregulated *BIRC2* in HeLa-S3 cells (Figure 3C,D). We further investigated the role of miR143-3p in cervical SCC tumorigenesis. When we overexpressed miR143-3p, HeLa-S3 cells exhibited a reduced capacity to form colonies relative to control cells. Conversely, the overexpression of anti-miR-143-3p increased the HeLa-S3 cells’ capacity to form colonies relative to that of control cells (Figure 3E).

### 2.5. Role of LC161 in Colony Formation

**Figure 4:**
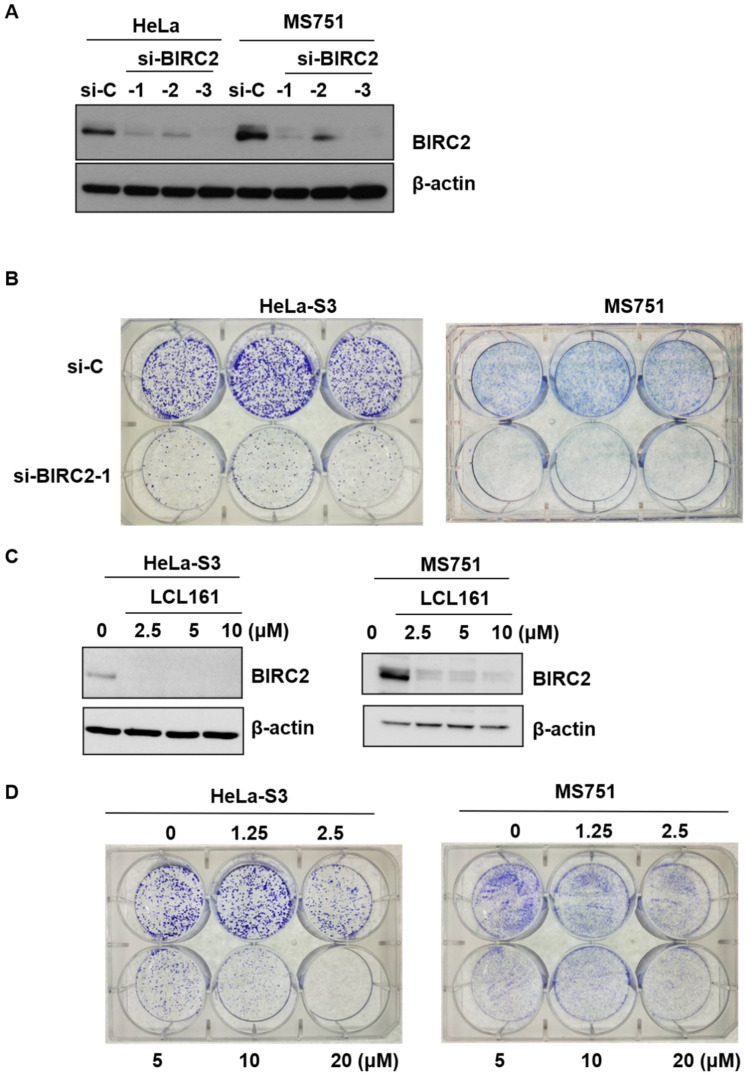
Effect of LCL161 on clonogenicity. (**A**) HeLa-S3 or MS751 cells were transiently transfected with nontargeting siRNA (si-control, si-C), BIRC2 siRNA (si-BIRC2-1, -2, -3) for 72 h. Cell lysates were subjected to Western blotting using antibodies raised against BIRC2 and β-actin. (**B**) HeLa-S3 or MS751 cells were treated with si-C and si-BIRC2-1 for 7–14 days. Colony formation was analyzed through the clonogenic assay. (**C**) HeLa-S3 or MS751 cells were treated with BIRC2 inhibitor LCL161 for 24 h and cell lysates were subjected to Western blotting using antibodies raised against BIRC2 and β-actin. (**D**) HeLa-S3 or MS751 cells were treated with vehicle or varying doses of LCL161 (1.25, 2.5, 5, 10, and 20 μM) for 7–14 days. Colony formation was analyzed through the clonogenic assay.

*BIRC2* expression was silenced by siRNA, which exhibited reduced protein expression (Figure 4A) and a lower capacity to form colonies relative to control cells (Figure 4B). A similar effect was elicited by LCL161, a SMAC mimetic that inhibits multiple inhibitors of apoptosis (IAPs) [15] in the protein expression of BIRC2 and the clonogenic capacity assay (Figure 4C,D, respectively).

### 2.6. Inhibition of BIRC2 Sensitizes Cervical Cancer to Topotecan-Mediated Cell Death and Cell Viability 

**Figure 5:**
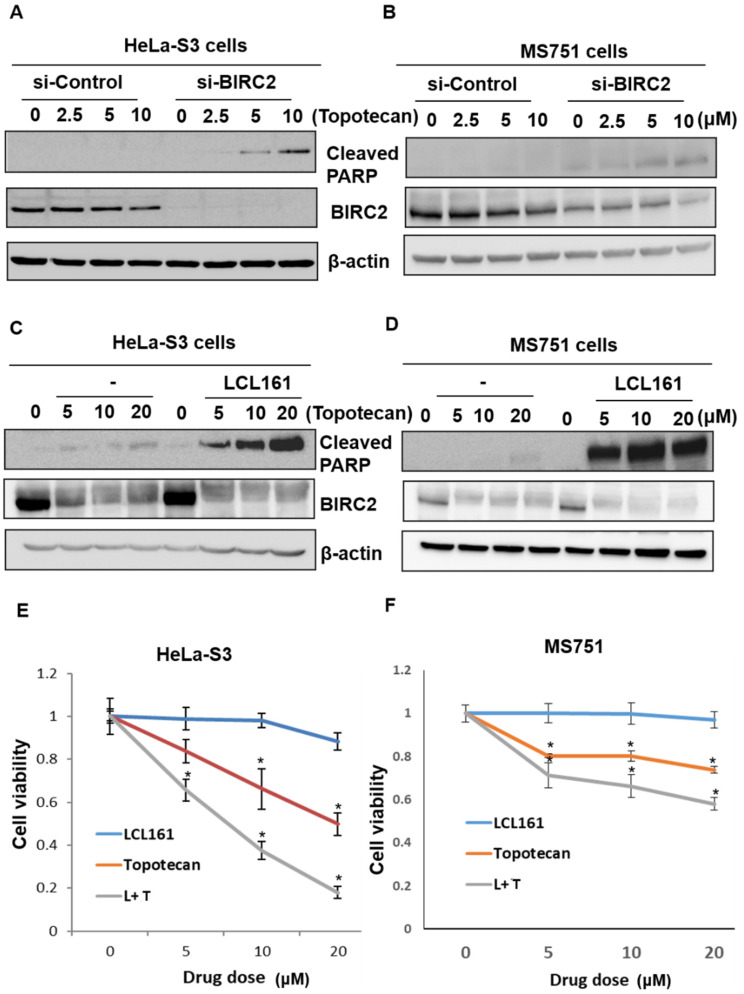
The combination of LCL161 and topotecan exerted synergistic effects on apoptosis and cell viability in cervical cancer cells. (**A**,**B**) HeLa-S3 or MS751 cells were transiently transfected with si-C or si-BIRC2 for 24 h following the addition of vehicle or varying doses of topotecan (2.5, 5, and 10 μM) for an additional 24 h. (**C**,**D**) HeLa-S3 or MS751 cells were treated with LCL161 (10 μM) for 24 h following the addition of vehicle or varying doses of topotecan (5, 10, and 20 μM) for an additional 24 h. Subsequently, cleaved PARP, BIRC2, and β-actin were analyzed through Western blotting. For normalization, β-actin served as the loading control. (**E**,**F**) HeLa-S3 or MS751 cells were treated with vehicle or varying doses of LCL161 (5, 10, and 20 μM), topotecan (5, 10, and 20 μM), or a combination of LCL161 and topotecan (L + T) for 24 h. Cell survival was analyzed through the MTT assay. * *p* < 0.05 relative to controls.

Because topotecan is a recommended first-line therapy [16], we examined the effect of *BIRC2* inhibition on cervical cancer cells treated with topotecan. In subsequent experiments, cleaved PARP was used as a marker of apoptosis [17]. HeLa cells contain HPV-18 sequences, and MS751 cells contain HPV 18 and a partial HPV45 genome. The viability of the cells after transfection with siRNA for nontargeting control (si-Control) or *BIRC2* (si-BIRC2) were 93 % and 89 % in HeLa-S3 cells (*p* = 0.343), and 87 % and 74 % in MS751 cells (*p* = 0.244), respectively. (Supplementary Figure S3). The treatment of HeLa-S3 or MS751 cervical cancer cells with si-BIRC2 or varying concentrations of topotecan did not significantly affect cell apoptosis. However, *BIRC2* knockdown significantly promoted cell apoptosis in cervical cancer cells relative to nontargeting siRNA (si-Control) after topotecan treatment (Figure 5A,B). Similarly, LCL161 significantly increased cleaved PARP activity after topotecan treatment relative to vehicle-treated cells (Figure 5C,D). We then investigated their potential synergistic effects. Figure 5E,F present the MTT assay results, which indicate that the combination of LCL161 and topotecan synergistically inhibited cell viability.

### 2.7. Synergistic Effects of LCL161 and Topotecan on Tumorigenesis in a Xenograft Tumor Model

**Figure 6:**
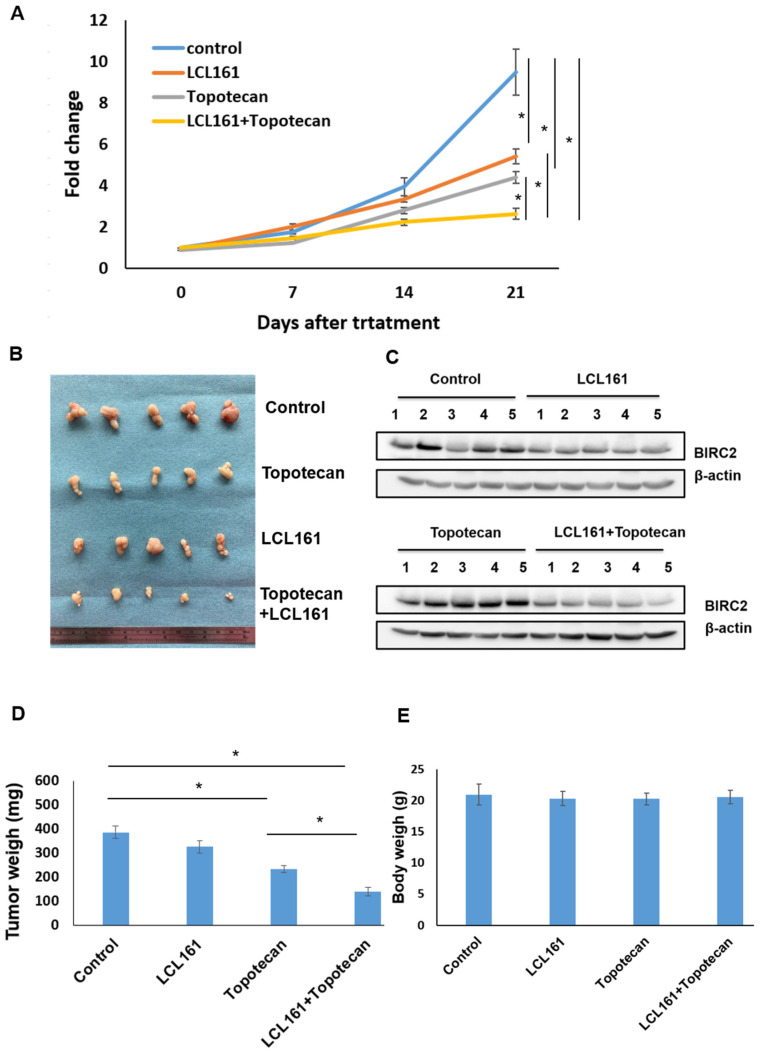
LCL161 and topotecan produced synergistic effects against tumor growth. (**A**) Inhibitory effect of the LCL161–topotecan combination against MS751 growth in a xenograft tumor model. (**B**) Representative images of tumors obtained through the inoculation of MS751 cells in nude mice under four therapeutic conditions (from top to bottom): control, LCL161 treatment, topotecan treatment, and LCL161–topotecan treatment. (**C**) Extracts from tumors exposed to LCL161, topotecan, and LCL161–topotecan were subjected to immunoblotting with antibodies raised against BIRC2 and β-actin and analyzed through Western blotting. For normalization, β-actin served as the loading control. (**D**,**E**) Effect of the LCL161–topotecan combination on the tumor weight and body weight of mice with xenografted cervical cancer cells. * *p* < 0.05 relative to controls.

Because the combination of LCL161 and topotecan promoted apoptosis in cervical cell lines, their synergistic effect was investigated using an in vivo xenograft tumor model. The experiments confirmed that the LCL161–topotecan combination produced synergistic effects against in vivo tumor growth (Figure 6A). Compared with the administration of either treatment alone, the combination led to the greatest antitumor activity in the xenograft model (n = 5 in each group; Figure 6B,D). *BIRC2* inhibition was more prominent when LCL161 was used in a xenograft tumor model (Figure 6C). The body weight of the mice did not change during treatment (Figure 6E).

### 2.8. BIRC2 Protein Expression in α7-HPV-Related Cervical SCC (Including Mixed Infections)

**Figure 7:**
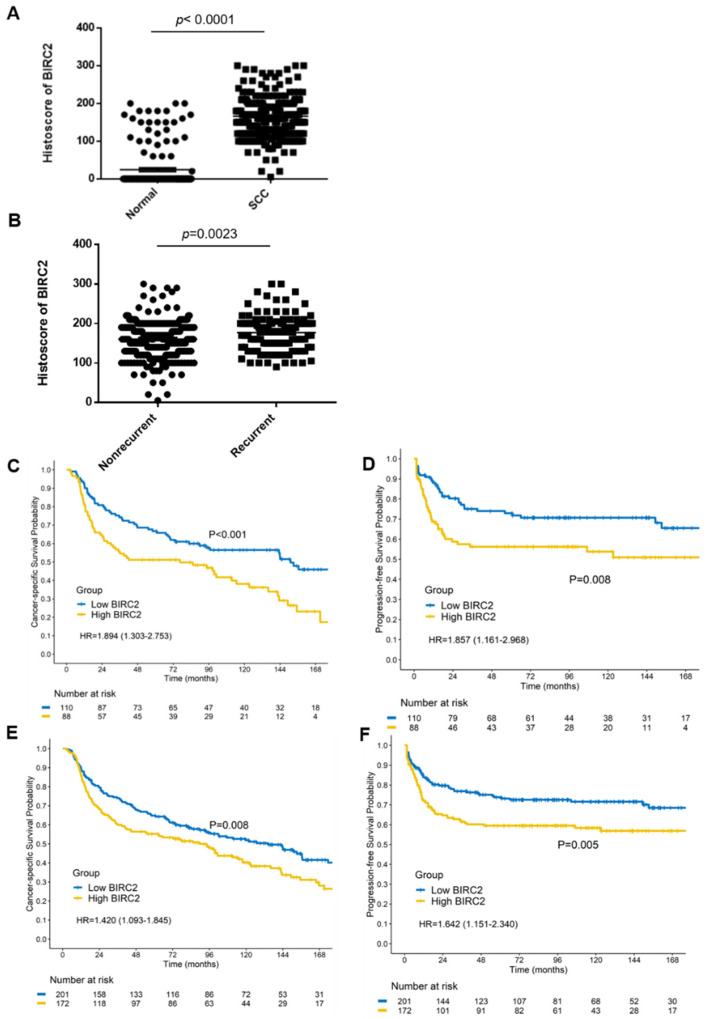
BIRC2 expression in α7-HPV-related cervical SCC (including mixed infections). (**A**) Immunohistochemical testing was conducted to analyze BIRC2 protein expression in formalin-fixed paraffin-embedded samples of biopsy tissue from patients with cervical SCC (n = 373) and adjacent normal tissues (n = 168). (**B**) Histoscores of cervical SCC were divided into recurrent (n = 124) and nonrecurrent tumors of cervical SCC (n = 249). (**C**) Kaplan–Meier survival curves indicate that for patients with α7-HPV-related cervical SCC in the pooled cohort (1993–2014), having a BIRC2 histoscore of ≥175 was associated with worse CSS (*p* < 0.001) and (**D**) PFS (*p* = 0.008). (**E**) Kaplan–Meier survival curves indicate that for patients with α7-HPV-related cervical SCC (including mixed infections) in the pooled cohort (1993–2014), having a BIRC2 histoscore of ≥175 was associated with significantly worse CSS (*p* = 0.008) and (**F**) PFS (*p* = 0.005).

The BIRC2 protein levels of patients with clinical cervical SCC were validated through immunohistochemical studies. As expected, BIRC2 histoscores were significantly higher in cervical SCC samples than in adjacent normal tissues in α7-HPV-related cervical SCC (*p* < 0.0001; Figure 7A). BIRC2 histoscores for cervical SCC with recurrent tumors (n = 124) were higher than those for cervical SCC with nonrecurrent tumors (n = 249; *p* = 0.0023; Figure 7B). However, among patients with α7-HPV-related cervical SCC who were treated between 2001 and 2014 (n = 117), those with BIRC2 histoscores of ≥175 had worse (but nonsignificant) CSS (*p* = 0.178) and PFS (*p* = 0.214; Supplementary Figure S4). Therefore, we applied IRB amendments and were approved to use the extended cohort of 1993–2000 and 2001–2014 was used to increase testing power. Subsequently, the 1993–2000 cohort was pooled with the 2001–2014 cohort with regard to α7-HPV-related cervical SCC (n = 198). Those with BIRC2 histoscores of ≥175 had worse CSS (HR = 1.89, 95% CI = 1.30–2.75; *p* < 0.001) and PFS (HR = 1.86, 95% CI = 1.16–2.97, *p* = 0.008; Figure 7C,D). When α7-HPV and mixed cervical SCC were included, patients with BIRC2 histoscores of ≥175 had worse CSS (HR = 1.42, 95% CI = 1.09–1.84, *p* = 0.008) and PFS (HR = 1.64, 95% CI = 1.15–2.34, *p* = 0.005; Figure 7E,F). As displayed in Supplementary Figure S5, BIRC2 histoscores were not significantly different (*p* = 0.7929) between nonrecurrent and recurrent non-α7-HPV-related cervical SCC (n = 45 and 32, respectively). These results suggest that BIRC2-mediated regulation is only relevant to α7-HPV-related cervical SCC (including multiple infections with coexisting non-α7-HPVs).

### 2.9. Univariate and Multivariate Analyses of Prognostic Factors

Table 1 presents the results of the univariate and multivariate analyses of prognostic factors. In both analyses, age, stage, HPV type (α7-HPV + α7-HPV-mixed vs. non-α7-HPV), and BIRC2 (histoscore of ≥175 vs. <175) were determined to be independent, significant prognostic factors.

## 3. Discussion

Studies have indicated that miR143-3p modulates drug resistance in various cancers [18,19]. Moreover, miR-143 is downregulated in cervical cancer and promotes apoptosis and inhibits tumor formation by targeting BCL-2 [13,20]. IAP antagonists are a class of compounds developed to induce cancer cell death by blocking the caspase inhibitory function of IAPs such as X-linked IAP and BIRC2. The aberrant expression of IAPs in human cancers is associated with chemoresistance. IAPs constitute promising molecular targets for therapeutic interventions designed to restore the ability of cancer cells to undergo apoptosis in response to radiotherapy. Studies have reported that *BIRC2* knockdown increased the sensitivity of mouse melanoma cells and breast cancer cells to immune checkpoint inhibitors [21,22]. 

The present study is the first to demonstrate that miR143-3p is downregulated in recurrent α7-HPV-related cervical SCC relative to nonpersistent or recurrent α7-HPV-related cervical SCC. Through the use of reporters, we confirmed that the miR-143 binding site on the 3′-UTR of BIRC2 was the main site responsible for directly suppressing *BIRC2* expression. Regarding clinical cervical SCC (n = 1837), we validated a previous finding that α7-HPVs (including mixed infections) are predictors of worse prognosis [7]. Moreover, BIRC2 (histoscore of ≥175 vs. <175), age, stage, and HPV type (α7-HPV + α7-HPV-mixed vs. non-α7-HPV) were implicated as independent significant prognostic factors through multivariate analysis.

*BIRC2* is a member of the antiapoptosis gene family, inhibiting apoptosis by interfering with the activation of caspases and SMAC mimetic compounds LCL161, birinapant (TL32711), and GDC-0152 were tested as anticancer agents in clinical trials [23]. 

In one study, several chemotherapeutic agents (e.g., paclitaxel, cisplatin and topotecan) were used against cervical SCC [24]. Clinical trials on patients with advanced solid tumors involving cotreatment with LCL161 and paclitaxel, as well as cotreatments with other chemotherapeutic agents, are ongoing [23,25]. Notably, studies have reported that a combination of LCL161 with cisplatin, vincristine, or immune checkpoint inhibitors produced the highest antitumor activity in cancer cells [26,27,28]. The combination of LCL161 with topotecan was tested for ovarian cancer [23]; however, this combination has yet to be tested for cervical cancer. Immunotherapies (particularly those involving immune checkpoint inhibitors) have demonstrated great potential in cervical cancer treatment, and multiple phase III trials are underway [29]. The KEYNOTE-826 trial verified the significant benefit of adding pembrolizumab to chemotherapy for metastatic/recurrent cervical cancer [30].

Herein, we demonstrated for the first time that high *BIRC2* expression is associated with significantly worse outcomes for α7-HPV-related cervical SCC (including multiple infections with α7-HPV and coexisting non-α7-HPVs). Further clinical studies of LCL161 combined with topotecan/cisplatin with or without the addition of immune checkpoint inhibitors for patients with α7-HPV-related cervical SCC are warranted, particularly when *BIRC2* aberrations are involved. 

In sum, our findings suggest that *BIRC2* is a novel prognostic factor and therapeutic target for α7-HPV-related cervical SCC (including mixed infections). Thus, protocols involving the combined administration of LCL161 with cisplatin-based chemotherapy and immune checkpoint inhibitors may facilitate the development of more effective chemoimmunotherapy options for patients with advanced or recurrent α7-HPV-related cervical SCC. 

## 4. Materials and Methods

### 4.1. Patients and Immunohistochemistry and Clinical Tissue Specimens

The study protocol was approved by an institutional review board (IRB; approval nos. IRB103-7267B and 202100402B0). Patients who received primary RT/CCRT for FIGO stages I–IV cervical SCC between 2001 and 2014 were retrospectively reviewed, with eligible patients selected for the discovery set. Those treated between 1993 and 2000 [7] were pooled to test whether the discovery set could be applied to a larger cohort. Tissue samples (FFPE tumor blocks) were available for patients with cervical SCC. Histology slides were reviewed and confirmed to indicate SCC of the cervix. Immunohistochemical testing was performed according to the protocol used in a previous study [31]. Briefly, 4-μm-thick tissues slices were deparaffinized in xylene and rehydrated through graded washes of ethanol in water. Sections were stained with the BIRC2 antibody by using a a BOND-MAX automated stainer (Leica Biosystems). Hematoxylin was used for counterstaining. A semiquantitative immunostaining score (histoscore) was calculated by multiplying the percentage of positive cells by their staining intensity (0 = negative, 1 = weak, 2 = moderate, 3 = strong). The minimum and maximum possible histoscores were 0 and 300 (i.e., 100% × 3), respectively.

### 4.2. HPV Genotyping

DNA was extracted from FFPE tissue or cervical swabs according to the protocol used in previous studies [2,7,8]. SPF1/GP6+ consensus primers were employed to amplify a fragment of approximately 184 base pairs in the L1 open reading frame. GAPDH was performed as an internal control. HPV genotyping was conducted as previously described by using an Easychip HPV Blot membrane (King Car, Yilan, Taiwan). In total, 38 types of HPVs (6, 11, 16, 18, 26, 31, 32, 33, 35, 37, 39, 42, 43, 44, 45, 51, 52, 53, 54, 55, 56, 58, 59, 61, 62, 66, 67, 68, 69, 70, 71 (CP8061), 72, 74, 81 (CP8304), 82 (MM4), 83 (MM7), 84 (MM8), and L1AE5) were detected in a single reaction, as noted in previous studies [2,7,8]. The α7-HPVs comprised HPVs 18, 39, 45, 59, 68, and 70. Mixed infections were defined as multiple infections with α7- and non-α7-HPVs.

### 4.3. RNA Extraction from FFPE

Five FFPE blocks constituting approximately 10 μm thick slices were selected and deparaffinized in a designated solution (QIAGEN, Hilden, Germany). RNA was extracted and DNase was treated using a miReasy FFPE kit (QIAGEN) according to the manufacturer’s instructions. RNA was quantified by using a bioanalyzer (Agilent Technologies, Palo Alto, CA, USA).

### 4.4. miRNA 4.0 Array 

Affymetrix miR 4.0 array analysis was conducted according to the manufacturer’s instructions [32]. This array contains 2578 human mature miR probe sets. Briefly, 1 μg of the total RNA of each sample (n = 6) was subjected to a tailing reaction that was labeled using the FlashTag Biotin HSR RNA Labeling Kit (Genisphere, Hatfield, PA, USA); the ligation of biotinylated signal molecules to the RNA sample was then performed according to the manufacturer’s instructions. Each sample was then hybridized to a 4.0 miR array at 48 °C for 16 h and then washed and stained using a Fluidics Station 450. After staining, the chip was scanned using the GeneChip Scanner 3000 7G. Expression levels of miR transcripts were captured through the probe set by using Command Console 3.2 (Affymetrix, Santa Clara, CA, USA). 

### 4.5. HTA 2.0

For HTA, the integrity of total RNA was measured using an Agilent 2100 Bioanalyzer (Agilent Technologies, Palo Alto, CA, USA). RNA was amplified using a GeneChip WT PLUS reagent kit (Affymetrix) according to the manufacturer’s instructions. Labeled targets were hybridized to HTA for 16 h at 45 °C and washed according to the standard Affymetrix protocols [33]. Transcript expression levels were captured through a probe set by using Command Console 3.2 (Affymetrix). Transcriptome Analysis Console software was used to analyze both HTA and miR 4.0 arrays.

### 4.6. Cell Lines and Culture

Human cervical cancer cell lines HeLa-S3 (ATCC CCL-2.2) and MS751 (ATCC HTB-34) were purchased from the American Type Culture Collection (Manassas, VA, USA) and cultured in Dulbecco’s modified Eagle’s medium supplemented with 10% (*v*/*v*) fetal bovine serum (FBS) and α-MEM medium containing 10% (*v*/*v*) FBS, respectively. 

### 4.7. Antibodies and Reagents

Anti-BIRC2 rabbit polyclonal antibody was obtained from GeneTex (San Antonio, TX, USA), β-actin was purchased from Santa Cruz Biotechnology (Santa Cruz, CA, USA); rabbit polyclonal antibodies against cleaved PARP was provided by Cell Signaling Technology (Danvers, MA, USA); and all chemicals were sourced from Sigma (St. Louis, MO, USA) unless otherwise indicated.

### 4.8. Plasmids

The pMir luciferase reporter vectors contained the 3′-UTR of BIRC2 (Luc-BIRC2-3′-UTR) that used the BIRC2-3′-UTR forward primer 5′-AGCTGAGCTCAGAAAAATAGTCTATATTT-3′ and the BIRC2 3′-UTR reverse primer 5′-AGCTGAGCTCAGCACTTTATTGAGATGTT-3′. The forward primer for mutant Luc-BIRC2-3′-UTR was 5′-agctGAGCTCAGCACTTTATTCTCT TGTT TCTCAC-3′. The PCR products were digested with *SacI* and ligated into the *SacI*/CIP-treated pMiR-REPORT miRNA Expression Vector System (Thermo Fisher Scientific, Waltham, MA, USA) according to a previously described protocol [10]. To establish a plasmid-based system for miR143-3p, the annealed oligonucleotides corresponding to a partial sequence were designed and ligated to pSuper.neo+GFP (pSuper). The cDNA sequence of the targeted mRNA region for miR143-3p genes—that is, the miR143-3p sequence—was 5′-UGAGAUGAAGCACUGUAGCUC-3′.

### 4.9. DNA Transfection

For the miR-143-3p overexpression experiments, pSuper-miR-143-3p or pSuper-scrambled were transfected into HeLa-S3 cells using Lipofectamine 2000 (Invitrogen) according to the manufacturer’s instructions. Transient transfection was performed for 48–72 h prior to cell harvest unless otherwise specified. For the anti-miR-143-3p experiments, anti-miR-143-3p or anti-scrambled duplexes were transfected into HeLa-S3 cells at a final concentration of 5 nM by using Lipofectamine RNAiMAX (Invitrogen) according to the manufacturer’s instructions. Transient transfection was conducted for 72 h prior to cell harvest unless otherwise specified. The anti-miR143-3p sequence was 5′-, and the anti-miR-143-3p sequence was 5′-GAGCUACAGUGCUUCAUCUCA-3′.

### 4.10. Reporter Gene Assay

For the Luc-BIRC2-3′UTR reporter assay, cell extracts were obtained by exposing cells to 1× reporter lysis buffer (Promega, Madison, WI, USA). Reporter/luciferase activity was measured using the luciferase assay reagent (Promega) after normalization to the corresponding β-galactosidase activity.

### 4.11. Western Blotting

The Western blotting protocol was described in a previous study [34]. Briefly, cells were harvested, washed twice with PBS, and lysed in ice-cold RIPA lysis buffer. Lysates were boiled in 1× SDS-sample buffer and resolved through SDS-PAGE. SDS-PAGE-separated proteins were transferred through electrophoresis onto a Hybond-PVDF membrane (Amersham Pharmacia Biotech, Piscataway, NJ, USA). Finally, blots were probed with the reported primary antibodies and appropriate secondary antibodies. 

### 4.12. RNA Extraction and Real-Time qPCR

Total RNA was extracted from cell lines using the TOOLSmart RNA Extractor (Biotools, Taipei City, Taiwan) according to the manufacturer’s instructions. Approximately 350 ng of the total RNA from each sample and the expression levels of miR-143-3p in both cell lines and FFPE tissues were analyzed using the TaqMan MicroRNA Assay (assay ID: 002249). The expression levels of hsa-miR-16 (TaqMan MicroRNA Assay ID: 000008) were used as an internal control. RNA samples were subjected to real-time qPCR. Transcription levels were normalized to the GAPDH values of each sample. The primer sequences were as follows: BIRC2, 5′-GGAGATGATCCATGGGTAGA-3′ (sense), 5′-ACAAACTCTTGGCCTTTCAT-3′(antisense); GAPDH: 5′-GGTATCGTGGAAGGACTCATGAC-3′ (sense), 5′-ATGCCAGTGAGCTTCCCGT-3′ (antisense); and 18S rRNA, 5′-AAACGGCTACCACATCCAAG-3′ (sense), 5′-CCTCCAATGGATCCTCGTTA-3′ (antisense). Amplification was performed as follows: initial denaturation (10 min) at 95 °C, followed by 45 cycles at 95 °C for 15 s and 45 cycles at 60 °C for 1 min using an ABI PRISM 7900 HT instrument (Applied Biosystems, Foster City, CA, USA). All measurements were performed in duplicate, and the threshold cycle Ct was determined.

### 4.13. Clonogenic Assays

The methodology used for clonogenic assays was described in a previous study [35]. Briefly, HeLa-S3 or MS751 cells were transfected with mir143-3p, siRNA for *BIRC2*, or LCL161 and seeded into 6-well dishes and maintained for 7–14 days. After fixing with 30% methanol/12.5% acetic acid, colonies were visualized using Brilliant Blue R Staining Solution. 

### 4.14. Cell Viability Assay

Approximately 1 × 10^4^ cells were seeded into each well of a 96-well culture plate for 24 h. Cells in serum-free media were treated with LCL161, topotecan, or a combination of LCL161 and topotecan. An MTT viability assay was then conducted by adding 25 μL of 5 mg/mL MTT into each well. After 4 h of incubation at 37 °C, the supernatant was discarded and 100 μL of DMSO was added to each well. The mixture was then shaken to dissolve the formazan, and absorbance was measured at 570 nm in a multiwell spectrophotometer (VICTOR 2; Perkin Elmer GMI, Ramsey, MN, USA) [33].

### 4.15. Animals and Treatment

Six-week-old female BALB/c nude mice were obtained from the National Laboratory Animal Center, Taipei City, Taiwan. The study protocol was reviewed and approved by the Animal Care Committee of the IRB of Chang Gung Memorial Hospital (approval no. 2019090301). MS751 cells were harvested, washed, and resuspended in Hanks’ balanced salt solution at 10^7^ cells/mL. Tumors were established through the subcutaneous inoculation of the cell suspension (100 μL) into the lateral hind leg of nude mice aged 6−8 weeks. When the average tumor size reached approximately 100 mm^3^, mice were randomized by tumor size to treatment groups: treatment with 10 mg/kg LCL161 and/or 2 mg/kg topotecan, both intraperitoneally injected 3 days a week. Specifically, the mice were randomized to four treatment arms (n = 5 per group) as follows: (1) vehicle, (2) LCL161 alone, (3) topotecan alone, and (4) a combination of LCL161 and topotecan. Over the course of the treatment, tumor growth was monitored on a weekly basis. Tumor volume (cm^3^) was calculated and taken as the in vivo proxy of tumor mass. At the end of the experiments, tumors were excised and extracted using RIPA buffer and then subjected to Western blotting according to the aforementioned protocol. For cell viability after siRNA knockdown experiments, approximately 1 × 10^6^ cells were seeded into 6 cm culture plate for 24 h and cells were transfected with si-Control or si-BIRC2 for 72 h Thereafter, they were trypsinized, stained, and counted for cell viability.

### 4.16. Statistical Considerations

PFS was defined as the time from the date of diagnosis to the date of cancer recurrence or progression or censored on the date of the final follow-up session. CSS was calculated from the time of diagnosis to the time of death from cervical cancer or the final follow-up session. The Kaplan–Meier method was employed to generate curves of estimated survival, and log-rank tests were conducted for curve comparison. The optimal cutoff of *BIRC2* expression was determined using log-rank statistics to yield the largest discrepancy in survival outcomes. Univariate and multivariate Cox proportional hazards regression with stepwise selection was performed to identify the risk factors for survival outcomes. Analyses were conducted using IBM SPSS Statistics for Windows, version 22.0 (IBM Corp., Armonk, NY, USA). Tests were two sided, and *p* values of <0.05 were regarded as statistically significant.

## Figures and Tables

**Table 1:** Univariate and multivariate analyses of prognostic factors.

		Univariate					Multivariate (Stepwise)		
Characteristics	N	HR	95% C.I.	*p*-Value			HR	95% C.I.	*p*-Value
Age	1837	1.02	1.02–1.03	<0.001			1.02	1.02–1.03	<0.001
FIGO Stage									
1	380	1(ref)					1(ref)		
2	888	1.32	1.11–1.58	0.002			1.35	1.13–1.62	0.001
3	448	2.31	1.91–2.79	<0.001			2.28	1.88–2.75	<0.001
4	121	4.68	3.64–6.02	<0.001			4.86	3.77–6.26	<0.001
Differentiation									
1	62	1(ref)							
2	775	1.18	0.83–1.69	0.361					
3	731	1.29	0.90–1.84	0.168					
Unknown	263	1.01	0.69–1.49	0.952					
BIRC2 (cut-off 175)									
Low BIRC2	220	1(ref)					1(ref)		
High BIRC2	229	1.35	1.06–1.72	0.016			1.37	1.07–1.76	0.012
Unknown	1388	1.09	0.90–1.32	0.368			1.40	1.03–1.90	0.032
HPV type									
NON-α7	1412	1(ref)					1(ref)		
α7+α7-mix *	425	1.16	1.01–1.34	0.034			1.40	1.08–1.82	0.012
HPV type									
NON-α7	1412	1(ref)							
α7	224	1.18	0.98–1.41	0.083					
α7-mix *	201	1.15	0.95–1.39	0.144					

FIGO: International Federation of Gynecology and Obstetrics. * α7-mix indicates multiple infections with at least one α7-HPV and at least one non-α7-HPV.

## Data Availability

The data presented in this study are available on request from the corresponding author.

## Supplementary Materials

The following are available online at https://www.mdpi.com/article/10.3390/ijms222011020/s1.
