# Supplementary material for: Inhibition of BIRC2 Sensitizes α7-HPV-Related Cervical Squamous Cell Carcinoma to Chemotherapy
Source: Int J Mol Sci. 2021 Oct 13;22(20):11020. doi: 10.3390/ijms222011020 (PMC8539319; doi:10.3390/ijms222011020)
Supplement: Supplementary file 1 [file ijms-22-11020-s001.zip › ijms-1392373-supplementary.pdf]

Supplementary Materials:

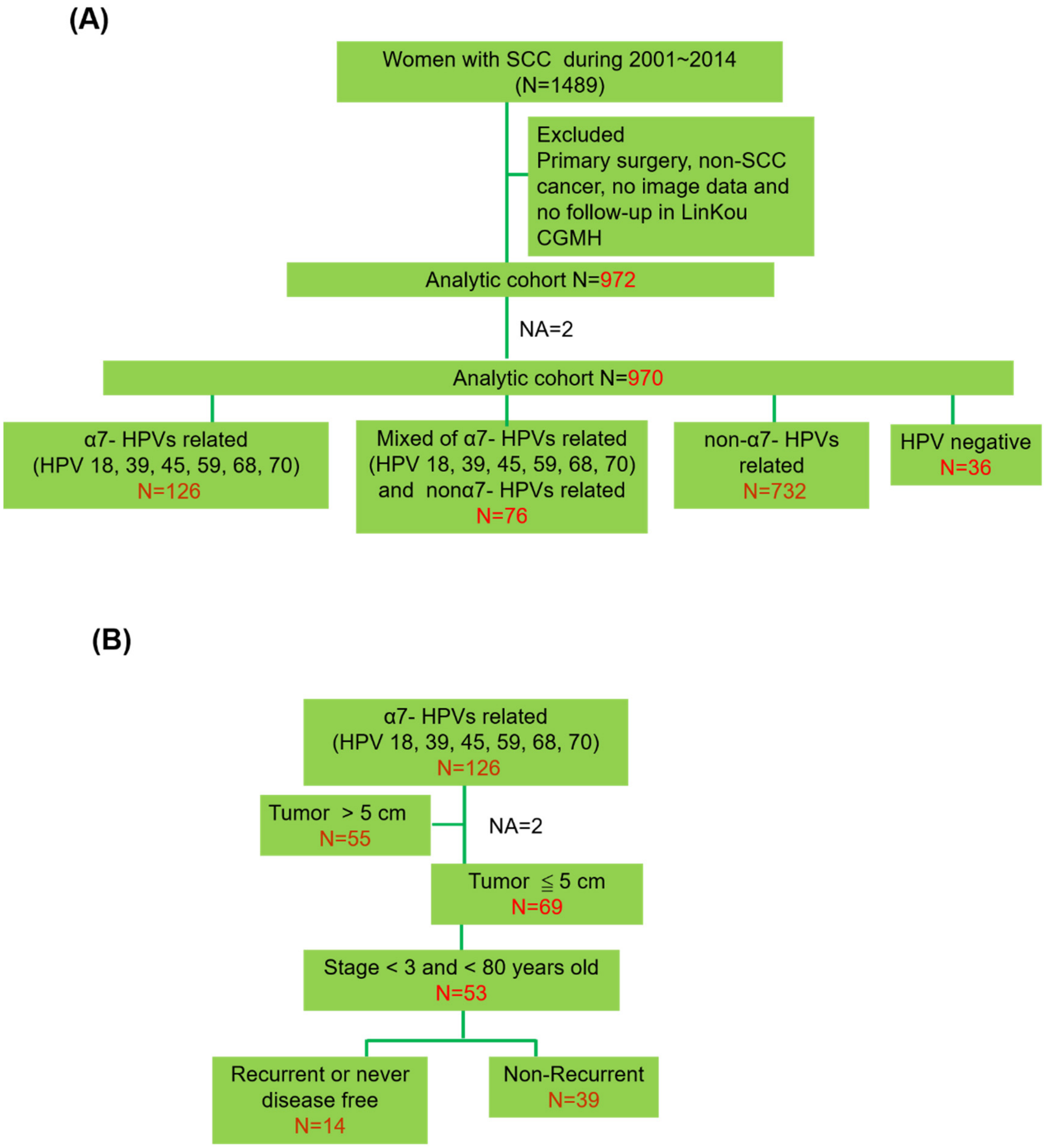

Supplementary Figure S1. A&B: Study flowchart.

A

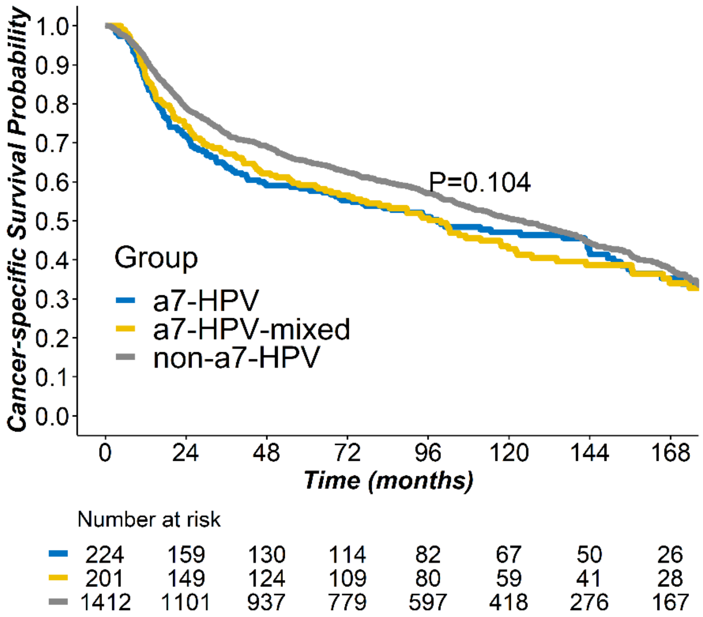

B

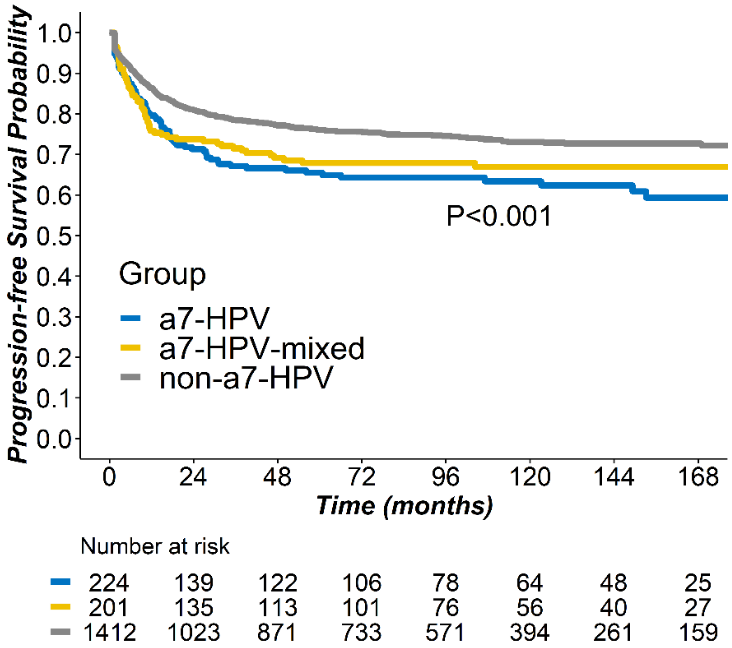

Supplementary Figure S2. Kaplan–Meier survival curves presenting **(A)** CSS and **(B)** PFS according to HPV genotype (α7-HPV, α7 HPV-mixed, or non-α7-HPV) in the 1993–2014 cohort.

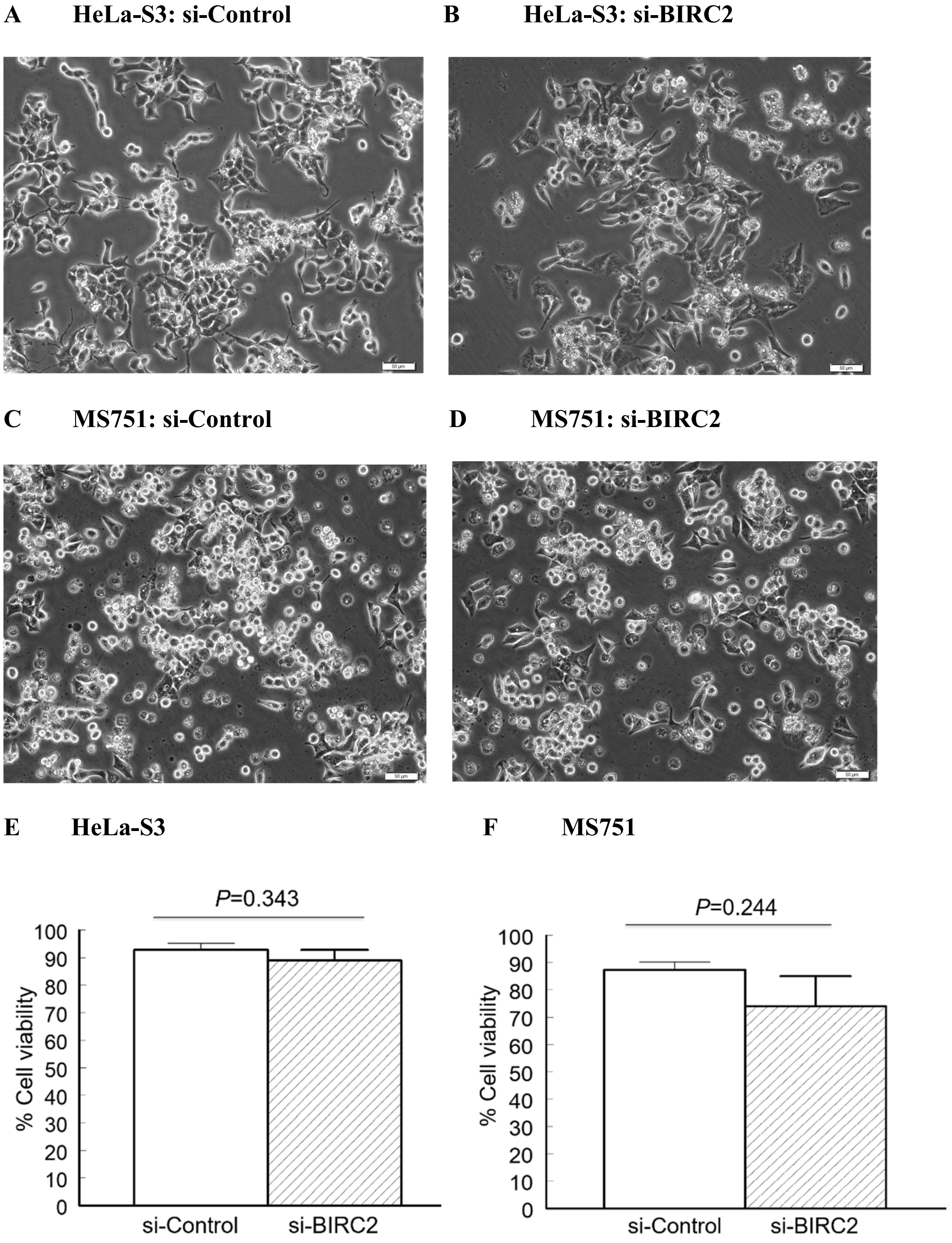

Supplementary Figure S3. Effects on cervical cancer cell morphology and cell viability after siRNA for control (si-Control) or BIRC2 (si-BIRC2) treatment. **(A~D)** Representative cell morphology images of HeLa-S3 or MS751 cells were subjected to control or BIRC2 silencing using a specific siRNA for 72 h. Scale bar, 50μm. **(E, F)** HeLa-S3 or MS751 cells were treated with si-Control or si-BIRC2 for 72 h. Cell viability quantitatively analyzed by the trypan blue assay. The viability of the cells after transfection with si-Control or si-BIRC2 was 93 % and 89 % for HeLa-S3, and 87 % and 74 % for MS751, respectively. In the quantitative bar graph, the results are expressed as mean ± standard error of the mean.

A

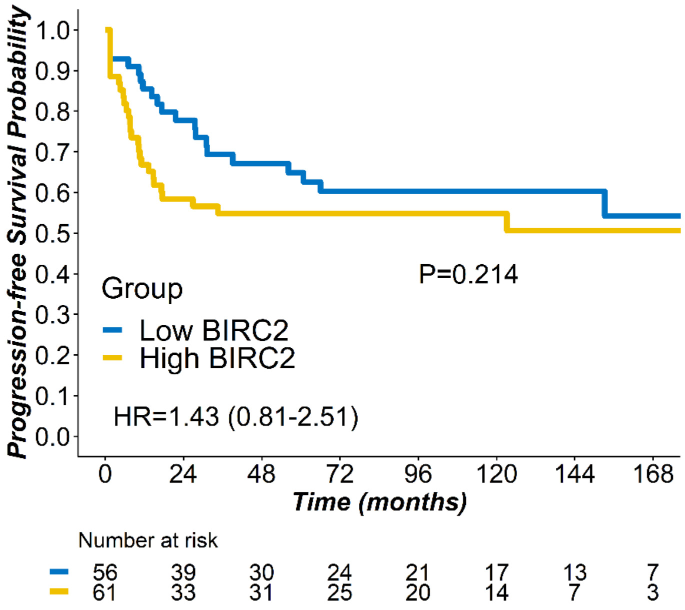

B

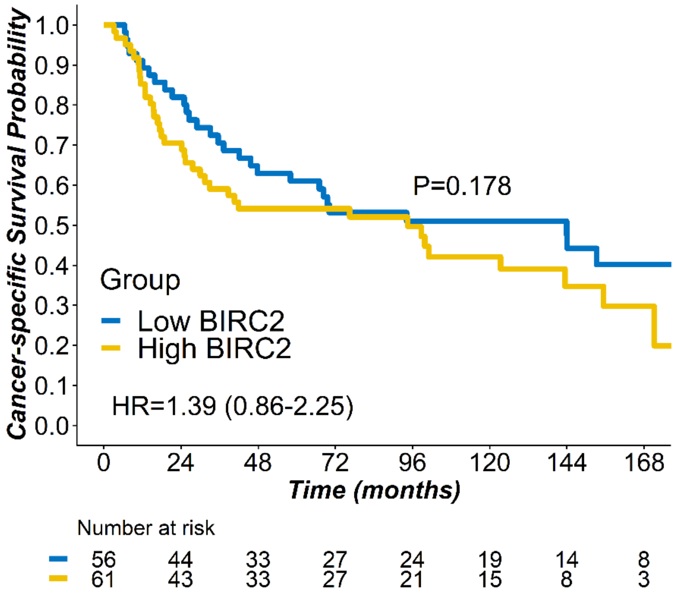

Supplementary Figure S4. Kaplan–Meier survival curves of **(A)** CSS and **(B)** PFS of α7-HPV related SCC in the 2001–2014 cohort according to BIRC2 histoscores (>175 vs. ≤ 175).

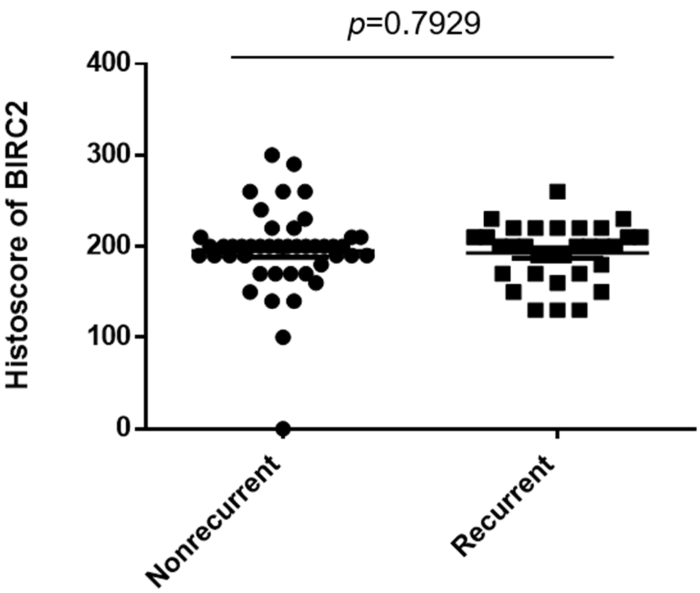

Supplementary Figure S5. BIRC2 histoscores in matched non-α7-HPV-related cervical SCC according to recurrence/progression status.
